# “Balancing” balancing selection? Assortative mating at the major histocompatibility complex despite molecular signatures of balancing selection

**DOI:** 10.1002/ece3.5087

**Published:** 2019-04-13

**Authors:** Joel W. G. Slade, Matthew J. Watson, Elizabeth A. MacDougall‐Shackleton

**Affiliations:** ^1^ Department of Biology University of Western Ontario London Ontario Canada

**Keywords:** assortative mating, balancing selection, major histocompatibility complex, positive selection, song sparrows, trans‐species polymorphism

## Abstract

In vertebrate animals, genes of the major histocompatibility complex (MHC) determine the set of pathogens to which an individual's adaptive immune system can respond. MHC genes are extraordinarily polymorphic, often showing elevated nonsynonymous relative to synonymous sequence variation and sharing presumably ancient polymorphisms between lineages. These patterns likely reflect pathogen‐mediated balancing selection, for example, rare‐allele or heterozygote advantage. Such selection is often reinforced by disassortative mating at MHC. We characterized exon 2 of MHC class II, corresponding to the hypervariable peptide‐binding region, in song sparrows (*Melospiza melodia*). We compared nonsynonymous to synonymous sequence variation in order to identify positively selected sites; assessed evidence for trans‐species polymorphisms indicating ancient balancing selection; and compared MHC similarity of socially mated pairs to expectations under random mating. Six codons showed elevated ratios of nonsynonymous to synonymous variation, consistent with balancing selection, and we characterized several alleles similar to those occurring in at least four other avian families. Despite this evidence for historical balancing selection, mated pairs were significantly more similar at MHC than were randomly generated pairings. Nonrandom mating at MHC thus appears to partially counteract, not reinforce, pathogen‐mediated balancing selection in this system. We suggest that in systems where individual fitness does not increase monotonically with MHC diversity, assortative mating may help to avoid excessive offspring heterozygosity that could otherwise arise from long‐standing balancing selection.

## 1 INTRODUCTION

In jawed vertebrate animals, the major histocompatibility complex (MHC) is a key component of adaptive immune defense. MHC genes encode cell‐surface glycoproteins that recognize and bind antigenic peptides, present them to T cells, and, in the case of nonself antigens, initiate an adaptive immune response (Trowsdale, 1995). MHC genotype determines the suite of pathogens to which an individual can respond, making these loci critical to disease resistance and subject to intense pathogen‐mediated selection. Although both directional (e.g., good genes, locally good genes) and balancing selection (e.g., rare‐allele advantage, heterozygote advantage) are well established to occur at MHC, the latter mode of selection is particularly well‐studied (Bernatchez & Landry, 2003). Supporting the widespread importance of balancing selection at MHC, these loci are the most polymorphic in the vertebrate genome: In some systems, upwards of a thousand alleles are maintained (Robinson, 2003).

Balancing selection at MHC may take the form of negative frequency‐dependent selection, arising from arms races with pathogens (Slade & McCallum, 1992), and/or heterozygote advantage (Niskanen et al., 2014). Contemporary balancing selection can be demonstrated directly, for example, if disease resistance is higher in individuals bearing rare alleles (Phillips et al., 2018) or highly heterozygous genotypes (Doherty & Zinkernagel, 1975; McClelland, Penn, & Potts, 2003; Penn, Damjanovich, & Potts, 2002). In addition to such snapshots of current selection regimes, historical balancing selection has also been inferred over longer timescales. Signatures of past balancing selection include the occurrence of trans‐species polymorphisms, thought to be maintained by long‐standing balancing selection that originated before the lineages became reproductively isolated (Hedrick, 1998; Hess & Edwards, 2002; Klein, 1980). Past balancing selection can also be reflected in positive selection, inferred from an excess of nonsynonymous relative to synonymous sequence variation within a population. Although positive selection is rare across most of the genome (Yang & Swanson, 2002), this pattern is observed relatively frequently at MHC (Hughes & Hughes, 1995; Piertney & Oliver, 2006), suggesting that parasite‐mediated balancing selection has often favored new and rare variants at these loci.

The survival advantage associated with particular combinations of alleles suggests that provided individuals can assess the genetic similarity of potential mates, sexual selection in the form of disassortative mating may further reinforce the balancing effects of pathogen‐mediated natural selection. Pathogen‐mediated balancing selection and disassortative mating are both expected to increase individual diversity (heterozygosity): the former by maintaining multiple alleles within the population, and the latter by increasing the likelihood of dissimilar parental genotypes coming together to produce offspring. To the extent that disease resistance varies positively with MHC diversity (e.g., Brambilla, Keller, Bassano, & Grossen, 2018; Hughes & Nei, 1989), disassortative mating at MHC should enhance offspring quality. Indeed, preferences for MHC‐dissimilar mates have been reported for many animal species (Kamiya, O'Dwyer, Westerdahl, Senior, & Nakagawa, 2014), including mammals (e.g., mice *Mus musculus domesticus; *Yamazaki et al., 1979), fish (e.g., brown trout *Salmo trutta; *Forsberg, Dannewitz, Petersson, & Grahn, 2007), reptiles (e.g., tuatara *Sphenodon punctatus*; Miller, Moore, Nelson, & Daugherty, 2009), seabirds (e.g., blue petrels *Halobaena caerulea*; Leclaire, Strandh, Mardon, Westerdahl, & Bonadonna, 2017), and passerine birds (e.g., savannah sparrows *Passerculus sandwichensis*; Freeman‐Gallant, Johnson, Saponara, & Stanger, 2002). Nonrandom mating at MHC is thought to be facilitated by olfactory cues, because MHC gene products affect not only pathogen resistance but also individual odor (Brennan & Zufall, 2006; Milinski et al., 2005).

Although disassortative mating at MHC is taxonomically widespread among vertebrate animals, it is not universal. Some species mate assortatively, rather than disassortatively, at MHC (mammals: European badgers *Meles meles* (Sin et al., 2015); wolves *Canis lupus* (Galaverni et al., 2016); amphibians: tiger salamanders *Ambystoma tigrina* (Bos, Williams, Gopurenko, Bulut, & Dewoody, 2009); passerine birds: house sparrows *Passer domesticus* (Bonneaud, Chastel, Federici, Westerdahl, & Sorci, 2006)). Assortative mating at MHC may provide material benefits to the choosy individual, such as reduced risk of infection with novel pathogens carried by MHC‐dissimilar immigrants (Lewis, 1998). Such assortative mating may also confer genetic benefits to offspring, for example, by reducing outbreeding depression in hybrid zones, reducing the disruption of coadapted sets of alleles (Roberts, 2009), and reducing susceptibility to autoimmune disorders associated with excessive individual diversity at MHC (Wegner, Kalbe, Kurtz, Reusch, & Milinski, 2003). Genetic benefits to assortative mating may be particularly salient in systems in which MHC is highly polygenic due to extensive duplication (Bollmer, Dunn, Whittingham, & Wimpee, 2010; Hess & Edwards, 2002; Minias, Pikus, Whittingham, & Dunn, 2018). In such systems, assortative mating may allow individuals to avoid the disadvantages of producing offspring with an excessive number of alleles at MHC. If so, rather than uniformly reinforcing MHC diversity, sexual selection may under some circumstances counteract—or “balance”—the effects of historical balancing selection.

Finally, some species appear to mate randomly with respect to MHC profiles. This pattern is particularly well‐documented in passerine birds (e.g., great reed warblers *Acrocephalus arundinaceus* (Westerdahl, 2004); common yellowthroats *Geothlypis trichas* (Bollmer, Dunn, Freeman‐Gallant, & Whittingham, 2012); great tits *Parus major* (Sepil et al., 2015), although not restricted to this group (e.g., brown bears *Ursus arctos*; Kuduk et al., 2014). Random mating at MHC, particularly among free‐living animals, could suggest that any genetic benefits associated with MHC‐mediated pairing may be outweighed by material benefits or other critical traits. Alternatively, this pattern might reflect sensory constraints and an inability to assess potential mates’ MHC profiles. Thus, naturally occurring patterns of mate choice are particularly illuminating for species with documented phenotypic cues of MHC genotype.

**Figure 1:**
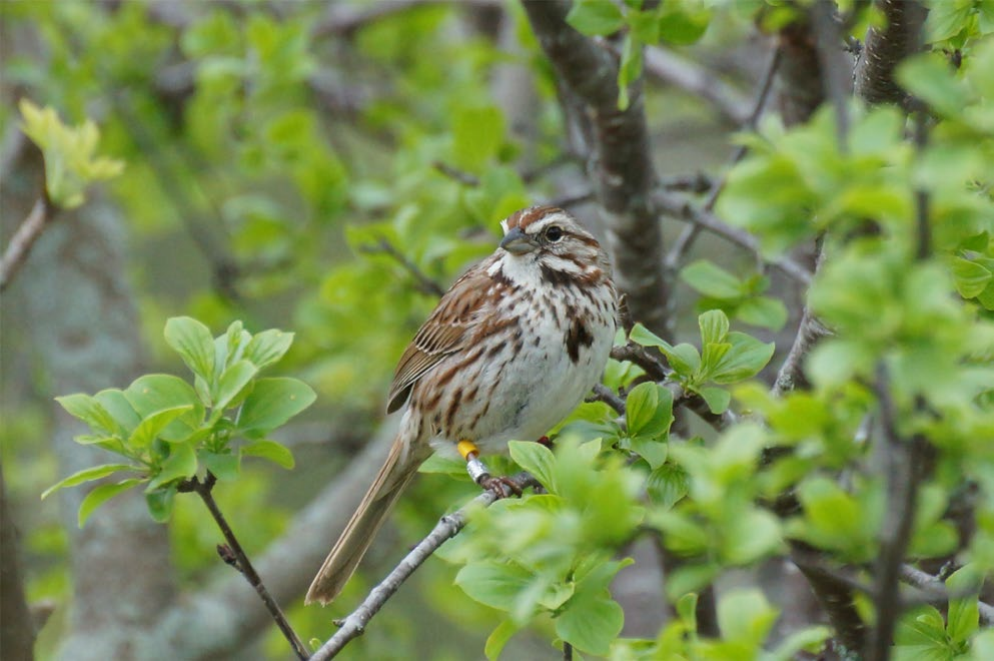
Song sparrow (*Melospiza melodia*). Photo credit: Tosha Kelly

We examined MHC‐mediated mate choice in free‐living song sparrows (*Melospiza melodia*; Figure 1). In the focal population, chemical composition of preen oil (the major source of body odor in birds) conveys information regarding MHC profiles (Slade et al., 2016). To our knowledge, song sparrows are the only passerine bird for which this relationship has been demonstrated. We then related patterns of social mate choice to evidence for historical pathogen‐mediated balancing selection, which we inferred from signatures of positive molecular evolution and trans‐species polymorphisms. MHC molecules are generally categorized into two major classes: Classes I and II interact primarily with intracellular (e.g., viruses) and extracellular antigens (e.g., bacteria), respectively (Klein, 1986). We focused on the hypervariable second exon of class II for two reasons. First, chemical composition of preen oil covaries with MHC class II genotype (Slade et al., 2016), raising the possibility that olfactory cues of class II similarity could facilitate nonrandom mating. Second, this region shows the highest individual‐ and population‐level variability in song sparrows (Slade, Sarquis‐Adamson, Gloor, Lachance, & MacDougall‐Shackleton, 2017) and other passerines (Minias et al., 2018). Song sparrows show as many as 26 alleles per individual at MHC class II, implying at least 13 loci (Slade, Sarquis‐Adamson, et al., 2017), and common yellowthroats as many as 39 alleles per individual, implying at least 20 loci (Bollmer et al., 2010).

To assess MHC‐mediated mating, we identified breeding pairs in the wild, calculated genetic distance between mates, and compared observed distances to those expected under random mating. To assess historical evidence of balancing selection, presumably mediated by pathogen resistance, we examined patterns of molecular evolution (i.e., ratios of nonsynonymous to synonymous variation), and surveyed for trans‐species polymorphisms by comparing song sparrow sequences at MHC class II to those from other passerine species. Ultimately, we sought to determine whether nonrandom mating at MHC affects individual diversity in a manner congruent with, or opposing, the inferred effects of past selection.

## 2 MATERIALS AND METHODS

### 2.1 Study animals and field methods

Fieldwork focused on a long‐term study population of migratory song sparrows breeding near Newboro, Ontario, Canada (44.6338°N, 76.3308°W). During spring 2014 (April 14‐June 2) and 2015 (April 13‐June 6), corresponding to nesting, egg‐laying, incubating, and provisioning offspring in the study population, we captured adult song sparrows; collected blood samples for genetic analysis; and identified socially mated pairs, using trapping records and behavioral observations as detailed below.

We captured song sparrows in two‐celled, seed‐baited Potter traps, which we checked once per hour, three times a day, between 07:00 and 11:00. We captured 69 birds in 2014 (44 males, 25 females) and 87 in 2015 (49 males, 38 females). These figures include 28 birds that were captured in both study years (25 males, three females); thus, our sample comprises 128 individuals (68 males, 60 females).

From each bird, we collected ~25 μl of whole blood via brachial venipuncture the first time it was captured each year. We blotted blood onto high wet‐strength filter paper saturated with 0.5 M Na‐EDTA (pH 8.0), allowed the blot to air‐dry, then stored it at room temperature awaiting DNA extraction. We identified sex based on the presence (male) or absence (female) of a cloacal protuberance, supplemented by unflattened wing length (measured with dial calipers to the nearest 0.1 mm). If not already banded, we outfitted the bird with a numbered aluminum leg band (Environment and Climate Change Canada Scientific Banding Permit 10691B), and a unique combination of three colored plastic leg bands to permit individual identification in the field. We recorded any other song sparrows in the trap (i.e., trapped in the other cell at the same time) and released birds at their site of capture. In almost all cases, birds were later resighted and/or recaptured, implying that they were resident breeders. Animal procedures were approved by the Animal Use Subcommittee at the University of Western Ontario (protocols 2008‐054 and 2015‐047 to EAM‐S) and conducted under the required federal permits.

Song sparrows are socially monogamous, and extrapair mating is rare in the study population: Fewer than 20% of nests contain extrapair offspring (Potvin & MacDougall‐Shackleton, 2009). For these reasons, and because high rates of nest failure make it challenging to collect blood from nestlings for genetic parentage analysis, we focused on social rather than genetic mate choice. We identified socially mated pairs through opportunistic behavioral observations on color‐banded individuals (e.g., copulations or copulation solicitations; female and male observed foraging together; female and male observed provisioning the same nest) combined with trapping records. We identified pairs based on trapping records if one or both of the following conditions were met: (a) Two individuals of opposite sex were trapped together at the same time (8.7% of all trapping records), or (b) two individuals of opposite sex were captured in the same trap within 48 hr of one another, and no other song sparrows were trapped at that location during the entire field season (8.8% of all trapping records). Based on these criteria, we identified 18 socially mated pairs in 2014 and 22 in 2015. These figures include one pair that remained the same over both field seasons; to avoid pseudoreplication, we included this pairing in the 2014 mate choice analysis only. These figures also include three individuals (one male, two females) that were each identified as part of a mated pair in 2014, but had a different mate in 2015: These were included in both years’ mate choice analyses.

### 2.2 Characterizing MHC

For each of the 128 birds sampled, we used polymerase chain reaction (PCR) to amplify the hypervariable second exon of class II MHC. We used primers *SospMHCint1f *(Slade et al., 2016) and *Int2r.1* (Edwards, Gasper, & March, 1998), which should bind within introns 1 and 2 respectively, to amplify exon 2. In addition to the priming sequence, each primer included a unique “barcode” sequence of eight base pairs; four “wobble” bases; and an adaptor sequence for the Illumina MiSeq platform. PCRs were conducted in a total volume of 30 μl, including 12.5 μl of GoTaq® Hot Start Master Mix (Promega), 0.2 μM of each primer, and 25–60 ng of template DNA. Cycling conditions were 3 min at 94°C; 28 cycles of 30 s at 94°C, 30 s at 62°C, and 45 s at 72°C; and a final extension step of 10 min at 72°C. We confirmed amplification by running a portion of each PCR product on a 2% agarose gel. We then pooled together all samples for a given field season (2014, 2015) to form a library. Each season's library was run on a separate Illumina MiSeq flow cell at the London Regional Genomics Center.

We used a pipeline (Gloor et al., 2010) together with individually unique combinations of barcode sequences to assign MHC reads to individuals. We identified and removed chimeric sequences using UCHIME (Edgar, Haas, Clemente, Quince, & Knight, 2011). We also filtered out any sequence occurring in fewer than 1% of an individual's total reads, as these rare sequences might represent errors in PCR or sequencing. This 1% threshold was established as described in Slade, Sarquis‐Adamson, et al. (2017); briefly, we used bacterial cloning (Promega pGEM‐T Easy Vector System) to generate colonies that each contained a single allele. We included these colonies on the flow cell run and used the frequency of secondary reads to establish the error rate associated with PCR and sequencing. After filtering, we aligned the remaining sequences in MEGA 7.0 (Kumar, Stecher, & Tamura, 2016). We trimmed each to a length of 216–219 base pairs (72‐73 codons; this variation in length reflects the presence of a 3‐bp indel), corresponding to codons 8‐80 of exon 2 of MHC class II (total length 90 codons; Minias et al., 2018) in order to restrict our analysis to the region of highest read quality and to maintain consistency with previous studies (Slade, Sarquis‐Adamson, et al., 2017; Slade et al., 2016; Slade, Watson, & MacDougall‐Shackleton, 2017). Hereafter, references to specific codon numbers (e.g., codon 2, codon 6) refer to positions within the trimmed region that we analyzed, not within the full exon 2. We confirmed similarity to other passerine sequences by querying the Basic Local Alignment Search tool (BLAST; Altschul, Gish, Miller, Myers, & Lipman, 1990) implemented in GenBank.

### 2.3 Mate choice analysis

To test whether song sparrows pair nonrandomly at MHC class II, we compared genetic distances between socially mated pairs to the randomized set of possible opposite‐sex combinations based on adults captured at the site during the corresponding field season. Because some allelic pairs are more similar in sequence than others, rather than simply quantify allele sharing, we used UniFrac (Lozupone & Knight, 2005) to take into account phylogenetic distances between alleles. Based on a phylogeny of all alleles detected, the algorithm calculates the distance between two individuals’ translated MHC repertoires, such that two individuals with the identical set of alleles would have a UniFrac distance of zero and two individuals with alleles derived from completely different clades in the reference tree would have a UniFrac distance of one (Lozupone & Knight, 2005). We first constructed a maximum‐likelihood phylogeny of all alleles recovered, over both field seasons, using Whelan and Goldman (2001) substitution with five discrete gamma categories. Based on this phylogeny, we calculated pairwise amino acid UniFrac distances for observed pairings (i.e., between socially mated pairs). Because genotype data were binary (i.e., presence or absence of an allele within an individual's genotype) rather than continuous, we calculated unweighted (qualitative) rather than weighted (quantitative) UniFrac. UniFrac distances were calculated using the package GUniFrac (Chen et al., 2012), implemented in R 3.4.0 (R Core Team, 2017).

We used a Monte Carlo simulation (Manly, 1997) implemented in a custom Microsoft Excel macro (Neff, Garner, Heath, & Heath, 2008) to generate 10,000 randomized male–female adult pairings. Genotypes from which randomized pairings were drawn were sorted by year, such that, for example, an adult female captured at the field site in 2014 was assumed to have had the potential to pair with any of the adult males present in that year. We then calculated pairwise genetic distance for each randomized pairing using unweighted UniFrac as described above. Finally, we calculated the average genetic distance over the 40 observed social pairings and compared this to the expected distribution of genetic distances based on random mating.

### 2.4 Sequence evolution

We tested for signatures of positive selection using all available genotypes from the study population in 2014 and 2015 (128 breeding adults). After filtering out chimeras and very low‐frequency reads as described above, we tested for positive selection across our sequenced portion of exon 2, using PAMLx 1.3.1 (Xu & Yang, 2013). Statistical significance was assessed by a *Z*‐test, bootstrapped at 500 replicates, and conducted in MEGA 7.0 (Kumar et al., 2016). To ensure we confidently identified positively selected sites, we tested for positive selection at each codon site separately using multiple complementary approaches implemented in datamonkey.org (Weaver et al., 2018) in addition to PAMLx 1.3.1. Specifically, we tested for positive selection using a mixed‐effects model of evolution (MEME; Murrell et al., 2012), fixed‐effects likelihood (FEL; Kosakovsky Pond & Frost, 2005), single‐likelihood ancestor counting (SLAC; Kosakovsky Pond & Frost, 2005), and fast, unconstrained Bayesian approximation (FUBAR; Murrell et al., 2013). Finally, because positive selection is expected to be strongest at peptide‐binding codons, we compared the set of codons our analyses identified as having experienced past positive selection to the set of codons identified as peptide‐binding in humans (Brown et al. 1993), as well as to those codons recently identified as positively selected in passerines in general (Minias et al., 2018).

In PAMLx 1.3.1, we used the *codeml* command (Yang, 2007) to calculate ω, defined as the ratio of nonsynonymous substitutions per nonsynonymous site (dN) to synonymous substitutions per synonymous site (dS). Codons (sites) at which ω is less than, equal to, or greater than one is interpreted as having undergone purifying (negative) selection (ω_0_,) neutral evolution (ω_1_), and positive selection (ω_2_), respectively. *Codeml* does not make a priori assumptions as to which codons are likely to experience each type of selection (Yang & Swanson, 2002).

To evaluate which model(s) of sequence evolution best explained the observed variation in MHC class II exon 2 sequence in *codeml*, we used an information theoretic approach (Burnham & Anderson, 2002), ranking candidate models generated by PAML based on Akaike's information criterion (AIC). Candidate models were M1a (nearly neutral; ω_0_ < 1, ω_1_ = 1), M2a (positive selection; ω_2_ > 1), M8 (positive selection with β distribution; 0 < ω < 1, ω > 1), and M7 (null model counterpart to M8). Models M1a and M2a use the ω distribution to model parameters, and models M7 and M8 use the β distribution, constrained to range between 0 and 1 (Yang, 2000). We were particularly interested in the explanatory power of M2a relative to M1a, and of M8 relative to M7, because models M2a and M8 permit some codons to experience positive selection, whereas the null models M1a and M7 assume that codons experience neutral evolution, but also test for purifying selection. We identified positively selected codons using the Bayes empirical Bayes approach (Yang, Wong, & Nielsen, 2005) implemented in PAMLx 1.3.1 (Xu & Yang, 2013).

### 2.5 Trans‐species polymorphisms

Trans‐species polymorphisms involve two or more alleles, each occurring in two or more species. We conducted a nonexhaustive survey for such polymorphisms, including variants that while not identical across species, are more similar to their heterospecific counterpart than to one or more conspecific alleles. We identified the ten most common alleles in the study population of song sparrows and queried them against BLAST in GenBank (Altschul et al., 1990). For each allele, we identified any heterospecific sequences with at least 94% DNA sequence similarity. In all, we retrieved 25 such sequences from other songbirds.

We constructed a maximum‐likelihood phylogeny of the ten most common song sparrow alleles and the 25 heterospecific sequences, using a Jukes–Cantor model. Interdigitation of song sparrow alleles with those of other species would suggest that allelic diversification occurred prior speciation events and that balancing selection is of ancient origin. Conversely, if the ten song sparrow alleles comprised a monophyletic clade, this would suggest that allelic diversification has occurred more recently than speciation events and that balancing selection is of relatively recent origin.

## 3 RESULTS

We identified 278 unique DNA sequences (i.e., differing at one or more base pairs) from the 128 individuals genotyped, with an average (± *SEM*) of 14.1 ± 0.2 alleles per individual. Number of alleles per individual did not differ between the two years of the study (unpaired *t* test, *t*_154_ = 1.42, *p = *0.16). Sequences have been deposited to GenBank (accession numbers KX263957‐KX264148; KX375230‐KX375341; MF197785‐MF197843; MH670952‐MH671105).

**Figure 2:**
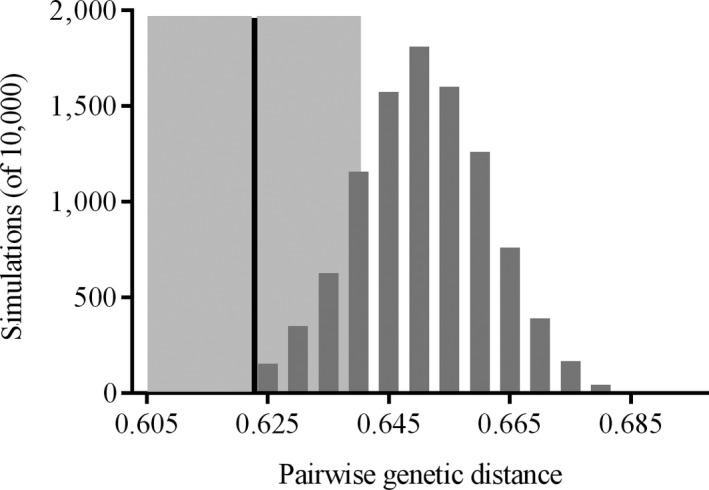
Frequency distribution of pairwise genetic distances at exon 2 of MHC class II, generated by Monte Carlo simulations of 10,000 randomized male–female pairings based on MHC genotypes characterized in the population. The vertical line at 0.623 denotes the average pairwise genetic distance between observed mates, inferred from behavioral and trapping records. Shading corresponds to ±1 *SE* around this average. Mated pairs were more similar at MHC than expected under random mating (two‐tailed *p* = 0.018)

Pairwise distance at MHC was lower, on average, for observed pairings (i.e., socially mated pairs; mean ± *SEM* = 0.623 ± 0.018) than for simulated pairings (0.650 ± 0.0001; two‐tailed *p* = 0.018; Figure 2). Thus, socially mated pairs were more similar at MHC than expected under random mating.

The observed pattern of molecular evolution, averaged across the sequenced portion of MHC class II exon 2, was consistent with positive selection (*Z*‐test, *Z* = 2.17, *p* = 0.016). Supporting this, of the four candidate models of codon evolution generated in PAML, the positive selection model (M2a) was the best supported, followed by the positive selection model with beta distribution (M8; Table 1); that is, both models allowing positive selection (i.e., M2a and M8) received comparable levels of support (AIC = 2) and were far better supported than models disallowing positive selection (i.e., M1a and M7; AIC ≥ 464, Table 1). The positive selection model (M2a) indicated that 94.3% of sites (68–69 sites) have experienced purifying selection, 4.8% (3–5 sites) have evolved through neutral drift, and 1% (~1 site) have experienced positive selection (Table 1). Similarly, the positive selection model with β distribution (M8) indicated that 99.1% (72–73 sites) of sites have experienced purifying selection, and 0.9% (~1 site) have experienced positive selection. Under M2a, the Bayes empirical Bayes approach identified ten codons with signatures of positive selection. Five of these have also been identified as positively selected in passerines by Minias et al. (2018), and three correspond to antigen‐binding sites in humans (Brown et al. 1993; Table 1). Under M8, the Bayes empirical Bayes approach identified fourteen codons with signatures of positive selection, seven of which matched sites in passerines (Minias et al., 2018), and six of which correspond to the antigen‐binding sites based on alignment with human sequence (Brown et al. 1993; Table 1). Generally consistent with the above findings, each of the alternative tests for positive selection implemented in Datamonkey 2.0 (Weaver et al., 2018) identified several codons showing the signature of positive selection (19, 11, 15, and 10 codons identified by MEME, FEL, SLAC, and FUBAR, respectively; Table 2). Across all tests for positive selection (i.e., PAML models M2a and M8; MEME, FEL, SLAC, and FUBAR tests in Datamonkey 2.0), six codons (2, 24, 42, 46, 62, and 73) were consistently identified by all methods as having undergone positive selection. Of these, codons 2 and 73 correspond to peptide‐binding sites in human MHC class II (Brown et al. 1993), and codons 42, 46, 62, and 73 have also been identified as positively selected across passerines in general (Minias et al., 2018).

**Table 1:** AIC‐ranked codon maximum‐likelihood models of sequence evolution, based on 518 DNA sequences of MHC class II, exon 2 recovered from song sparrows

Model	lnL	AIC	∆AIC	Parameter estimates	Positively selected codons
Positive selection ω (M2a)	−11,072	22,126	‐‐	*p*_0_ = 0.945, *p*_1_ = 0.046, *p*_2_ = 0.009 *ω*_0_ = 0.086, *ω*_1_ = 1, *ω*_2_ = 3.10	1, 2, 24, 26, 42*, 46*, 56, 60*, 62*, 73*
Positive selection β (M8)	−11,105	22,220	94	*p*_0_ = 0.959, *p*_1_ = 0.041, *p* = 0.041, q = 4.61, *ω* = 2.25	1, 2, 19*, 24, 26, 27*, 42*, 46*, 56, 60*, 62, 63*, 67, 73*
Nearly neutral (M1a)	−11,290	22,588	462	*p*_0_ = 0.977, *p*_1_ = 0.023, *ω*_0_ = 0.049, *ω*_1_ = 1	Not allowed
β (M7)	−11,359	22,722	596	*p* = 0.021, q = 0.164	Not allowed

Note

The estimated proportion of sites subject to purifying selection, neutral evolution (drift), and positive selection are denoted by *p*_0_ *, p*_1_, and* p*_2_ respectively. Underlining denotes codons that correspond to antigen‐binding sites in humans (Brown et al. 1993); asterisks denote codons that are positively selected in passerines in general (Minias et al., 2018).

**Table 2:** Positively selected sites indicated by mixed‐effects model of evolution (MEME), fixed‐effects likelihood (FEL), single‐likelihood ancestor counting (SLAC), and a fast, unconstrained Bayesian approximation for inferring selection (FUBAR)

Test	Positively selected sites
MEME	2, 7*, 17, 19*, 24, 25, 27*, 41, 42*, 46*, 49*, 50, 56, 60*, 62*, 66, 67, 71, 73*
FEL	2, 7*, 19*, 24, 42*, 46*, 56, 60*, 62*, 67, 73*
SLAC	2, 7*, 17, 19*, 24, 25, 27*, 41, 42*, 46*, 49*, 59*, 62*, 67, 73*
FUBAR	2, 7*, 19*, 24, 27*, 42*, 46*, 62*, 67, 73*

Note

Underlining denotes codons that correspond to antigen‐binding sites in humans (Brown et al. 1993); asterisks denote codons that are positively selected in passerines in general (Minias et al., 2018).

**Figure 3:**
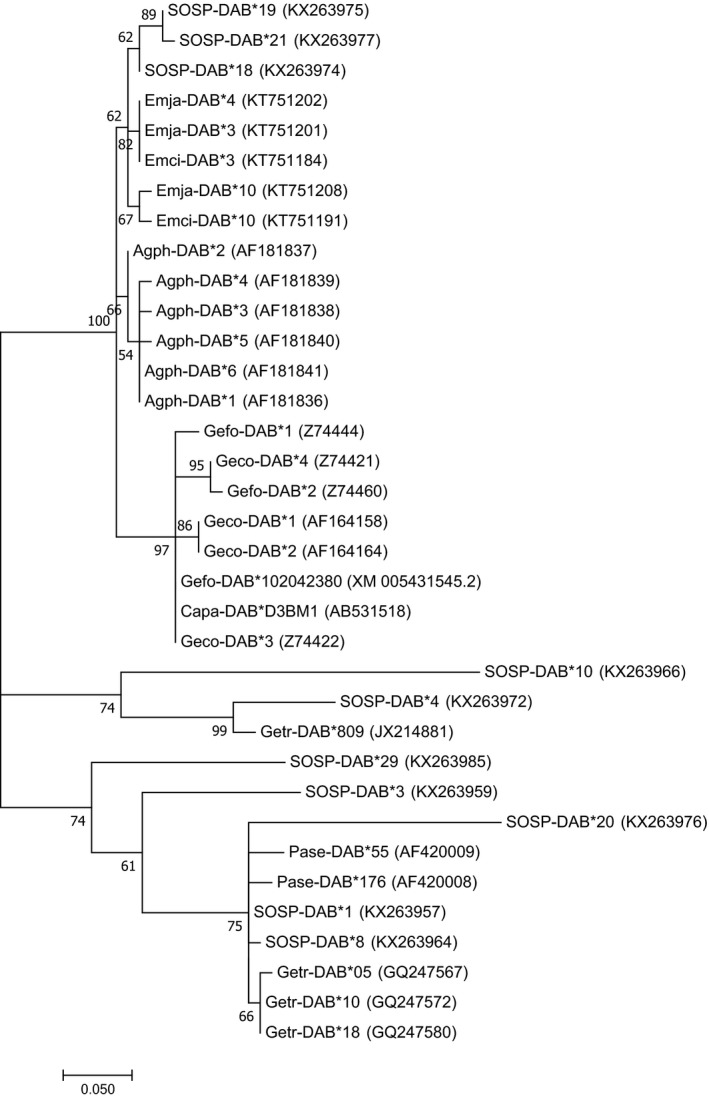
Unrooted phylogenetic tree of the ten most common alleles at MHC class II, exon 2 in song sparrows in this study (SOSP‐DAB*), plus 25 other sequences retrieved from GenBank with 94%–98% identity published for other songbird species. These additional sequences are denoted as Emja (Jankowski's bunting, *Emberiza jankowskii*), Emci (meadow bunting, *Emberiza cioides*), Agph (red‐winged blackbird, *Agelaius phoeniceus*), Gefo (medium ground finch, *Geospiza fortis*), Geco (Española cactus finch, *Geospiza conirostris*), Capa (woodpecker finch, *Cactospiza pallida*), Getr (common yellowthroat, *Geothlypis trichas*), and Pase (Savannah sparrow, *Passerculus sandwichensis*). All sequence names are followed by their GenBank accession number. Phylogeny was inferred by maximum likelihood based on the Jukes–Cantor model (log likelihood = −905.29). Bootstrap values, or the percentage of trees in which the associated alleles clustered together, are shown left of the nodes. Tree is drawn to scale, with branch lengths measured in the number of substitutions per site

The ten most common MHC class II alleles recovered from song sparrows showed 94%–98% sequence similarity to 25 sequences from eight other songbird species, belonging to five different families (Passerellidae: savannah sparrow *Passerculus sandwichensis*; Thraupidae: large cactus finch *Geospiza conirostris*, medium ground finch *G. fortis*, woodpecker finch *Cactospiza pallida*; Parulidae: common yellowthroat *Geothlypis trichis*; Icteridae: red‐winged blackbird *Agelaius phoeniceus*; Emberizinae: meadow bunting *Emberiza cioides*, Jankowski's bunting *E. jankowskii*; Figure 3). None of the 35 alleles investigated had 100% sequence identity to others published to GenBank; thus, we found no allelic pairs in song sparrows that were shared at 100% sequence similarity by another species. However, song sparrow alleles did not cluster as a single monophyletic clade (Figure 3). Instead, we observed several well‐supported clades in which one or more song sparrow alleles were more similar to heterospecific alleles within the clade than to conspecific alleles outside the clade (Figure 3). For example, song sparrow alleles SOSP‐DAB*18, *19, and *21 were more similar to alleles from Jankowski's bunting, meadow bunting, red‐winged blackbird, and the three species of Galapagos finch than to any other song sparrow alleles. Similarly, song sparrow allele SOSP‐DAB*4 was more similar to the common yellowthroat allele Getr‐DAB*809 than to any other song sparrow allele. Reciprocally, Getr‐DAB*809 was more similar to SOSP‐DAB*4 than to a different common yellowthroat allele in the phylogeny (Figure 3).

## 4 DISCUSSION

The diversifying effects of disassortative mating, that is, increasing genetic diversity of individuals and maintaining multiple alleles within populations, are similar to those of heterozygote advantage and negative frequency‐dependent selection. The general prevalence of balancing selection at MHC (Hedrick, 1998; Piertney & Oliver, 2006), combined with the discovery that these loci affect not just disease resistance but also odor, thus providing a plausible mechanism for nonrandom mating (Yamazaki et al., 1979), led to the reasonable initial expectation that when mating is nonrandom with respect to MHC, it should be disassortative such that mate choice operates in concert with the diversifying effects of balancing selection mediated by pathogens.

As predicted, we found evidence of historical balancing selection at MHC class II in our study population of song sparrows, presumably reflecting long‐standing evolutionary interactions with pathogens. Several codons showed an excess of nonsynonymous relative to synonymous variation, indicating positive Darwinian selection consistent with balancing selection. Likewise, many of the alleles characterized in song sparrows were more similar to alleles found in other species (including some from different avian families) than to other song sparrow sequences: This pattern implies long‐term balancing selection at MHC class II, which should promote the maintenance of multiple alleles at these loci and thus be associated with high levels of individual genetic diversity. In contrast, however, free‐living song sparrows paired assortatively rather than disassortatively at these loci, a pattern which should reduce individual genetic diversity of the resultant offspring. Indeed, nestling song sparrows in this population are less diverse at MHC class II than are adults (Watson, 2017), a pattern which might result from assortative mating. Collectively, our findings show that even when balancing selection is operating at MHC, nonrandom mating does not necessarily reinforce these diversifying effects.

Song sparrows in the study population showed several signals of past balancing selection. Although most codons appear to have been primarily subject to purifying selection, as is typical for functional coding loci (Yang & Swanson, 2002), six codons were consistently identified across multiple tests for sequence evolution as being likely to have experienced positive selection. Two of these, based on alignment to human MHC sequence, correspond to antigen‐binding positions that are likely to experience particularly intense selection (Hughes & Hughes, 1995). Moreover, four of the six codons identified correspond to the consensus of positively selected sites across passerines (Minias et al., 2018). The remaining positively selected codon (site 24) did not correspond to an antigen‐binding position based on sequence alignment with human MHC, nor was it reported to be positively selected site across passerines in general (Minias et al., 2018). Positive selection at MHC is widespread: In a recent review of the literature (25 publications on 25 vertebrate species), positive selection within the peptide‐binding region of MHC was reported in every study (Slade, 2018). Although both balancing and directional selection can generate positive selection (Hedrick, 2007), positive selection at MHC is generally considered to result from balancing selection (i.e., new alleles entering a population through mutation or immigration tend to increase in frequency). Still, we do not conclusively rule out the possibility that the observed positive selection could reflect a transient excess of nonsynonymous variants resulting from directional rather than balancing selection.

Further support for the importance of balancing selection in shaping variation at the song sparrow MHC class II comes from comparing sequences in song sparrows to their homologues in other songbird species. While we did not observe trans‐species polymorphisms as strictly defined, several alleles found in song sparrows were more similar to heterospecific alleles than to other conspecific alleles. For example, song sparrow sequences SOSP‐DAB*18, *19, and *21 were more similar to sequences from Thraupidae, Icteridae, and Emberizinae than to other alleles from song sparrows (family Passerellidae), despite the long‐standing divergence between Passerellidae and these other avian families (16.3–32.6 MY; Selvatti, Gonzaga, & de Moraes Russo, 2015). Although we cannot conclusively exclude the possibility of convergent sequence evolution, we think it probable that long‐standing balancing selection has maintained these allelic lineages since before the divergence of Passerellidae.

The expectation that sexual selection should favor MHC‐disassortative mating stems from the premise that offspring fitness (specifically, resistance to infectious disease) increases with increasing diversity at MHC. Clearly, offspring resulting from more MHC‐dissimilar pairings should be more diverse at MHC, and presumably capable of responding to a wider diversity of antigens (Klein, 1986). However, whether this translates into enhanced lifetime reproductive success, or even into superior disease resistance, is less clear. First, trade‐offs between nonadditive (compatible gene) effects, such as heterozygote advantage, and additive (good gene) effects may generate stabilizing selection on MHC diversity. Thus, given a finite level of gene product, excessive diversity at MHC may dilute the protective effects of locally good alleles (de Boer & Perelson, 1993; Kubinak, Nelson, Ruff, & Potts, 2012; Wegner et al., 2003). Second, the risk of parasitic infection associated with insufficient diversity at MHC may trade off with the risk of autoimmune disorders associated with excessive diversity (Apanius, Penn, Slev, Ruff, & Potts, 1997; Bottazzo, Todd, Mirakian, Belfiore, & Pujol‐Borrell, 1986; Wegner et al., 2003). Finally, pairings that are maximally dissimilar at MHC may generate outbreeding depression in offspring by disrupting coadapted gene complexes at MHC or other linked loci (Tregenza & Wedell, 2000). This risk may be magnified in hybrid zones, or when two or more locally adapted populations come into contact (Neff, 2004). We suggest that systems in which MHC is highly polygenic and polymorphic, such as song sparrows, are also likely to experience stabilizing rather than directional selection on MHC diversity. Optimal mate choice thus requires resolving trade‐offs between the benefits and the costs of high MHC diversity, which promote disassortative and assortative mating, respectively.

Our study did not address directly whether pathogen‐mediated balancing selection is currently operating in this population, for example, by comparing disease resistance of more versus less diverse genotypes. However, a cross‐sectional analysis in the study population showed that adults were more diverse than nestlings (more alleles per individual) at MHC class II (Watson, 2017). One interpretation of this pattern is that MHC‐diverse individuals are more likely to survive to adulthood than their less diverse counterparts. However, this pattern was observed in just one of two years of study, and MHC diversity did not predict overwinter return rates (interpreted as survivorship; Watson, 2017). Thus, if MHC diversity confers a survival advantage in this population, it does not do so consistently every year (Watson, 2017). Moreover, as noted above, assortative mating at MHC could also explain the difference in diversity between age cohorts. Song repertoire size, a sexually selected trait associated with early‐life condition, is also highest in males with intermediate rather than maximal MHC class II diversity in this population (Slade, Watson, et al., 2017). Thus, the relationship between MHC class II diversity and fitness in our study population does not appear to be uniformly positive, perhaps because of high standing levels of genetic variation.

We interpret the observed pattern of MHC‐assortative pairing as reflecting preferences, actively expressed by one or both sexes, for MHC‐similar social mates. In theory, assortative mating could also be explained through passive mechanisms, that is, by highly restricted natal dispersal such that close relatives are more likely to interact than nonrelatives. However, song sparrows are highly mobile, with natal dispersal distances on the order of 6 km (Zink & Dittmann, 1993) and individuals in our study population routinely migrating hundreds of kilometers between breeding and wintering grounds (Kelly et al., 2016). Thus, we do not think it likely that mobility constrains social mate choice at the geographic scale we investigated (<1 km^2^). Moreover, based on band‐recapture analysis, natal philopatry is low (5%–15% of new recruits each year were banded as nestlings; Stewart & MacDougall‐Shackleton, 2008) and strongly male‐biased, further reducing the likelihood of close relatives encountering one another as potential mates. Similarly, we cannot exclude the possibility that the observed mating patterns reflect active inbreeding, such that individuals preferentially mate with close relatives, which would also result in mated pairs being relatively similar at MHC. However, we think this unlikely as song sparrows do not appear to either prefer or avoid close relatives as social mates (Keller & Arcese, 1998). Behavioral tests under controlled conditions, for example, testing preferences for preen oil odor from MHC‐similar versus MHC‐dissimilar individuals (Leclaire et al., 2017), represent a critical next step to confirm (a) whether assortative pairing observed in the field reflects preferences expressed in the laboratory, and (b) whether chemical cues provide a mechanism for assessing MHC similarity in songbirds, as they do in other vertebrates (Brennan & Zufall, 2006; Leclaire et al., 2017; Milinski et al., 2005). Comparing the pairing behavior of free‐living animals to preferences expressed under standardized conditions will also shed light on potential trade‐offs between direct and indirect benefits associated with social and genetic mating decisions, and whether MHC‐related preferences are similar in both sexes.

Regardless of whether assortative mating stems from expressed mating preferences or from some other mechanism, its effects on individual genetic diversity (i.e., increasing homozygosity) oppose those of balancing selection (i.e., increasing divergence). In systems where strong and long‐standing balancing selection, presumably mediated by past arms races with pathogens, has generated high variation at immune loci, the ability of mate choice to “balance” balancing selection (i.e., to avoid producing offspring with an excessive number of different alleles) may be advantageous. Whether the assortative mating we observed in this population at the hypervariable class II MHC also occurs at the less variable class I MHC remains an open, and important, question. In particular, whereas song repertoire size does not increase monotonically with class II diversity in this population (Slade, Watson, et al., 2017), resistance to hematozoan infection does increase monotonically with class I diversity (Slade, Sarquis‐Adamson, et al., 2017).

Assortative mating does not necessarily reduce genetic diversity at the population level, at least under monogamous mating systems where the ability to attract a mate does not differ appreciably between genotypes. Assortative mating at MHC may thus balance the costs and benefits of genetic diversity within individuals, without constraining the evolutionary potential of populations to adapt to future changes in pathogen regime.

## CONFLICT OF INTEREST

The authors declared that they have no conflict of interests.

## AUTHOR CONTRIBUTIONS

JWGS and EAM‐S designed the experiment. JWGS, EAM‐S, and MJW collected field data. JWGS and MJW performed DNA extractions, PCR, and bioinformatics. JWGS performed statistical analyses. JWGS and EAM‐S prepared the manuscript.

## Data Availability

Data available from the Dryad Digital Repository: https://doi.org/10.5061/dryad.9v2362d. DNA sequences have been submitted to GenBank (accession numbers KX263957‐KX264148; KX375230‐KX375341; MF197785‐MF197843; MH670952‐MH671105).
